# Hemophagocytic Lymphohistiocytosis Induced by Severe Pandemic Influenza A (H1N1) 2009 Virus Infection: A Case Report

**DOI:** 10.1155/2011/951910

**Published:** 2011-04-14

**Authors:** Xiang-Yan Zhang, Xian-Wei Ye, Duan-Xing Feng, Jing Han, Dan Li, Cheng Zhang

**Affiliations:** ^1^Department of Respiratory Medicine, The People's Hospital of Guizhou Province, No. 83 Zhongshan Dong Road, Guiyang 550002, China; ^2^Academic Department, Guizhou Institute of Respiratory Diseases, Guiyang 550002, China

## Abstract

After early outbreaks in North America in April 2009, the pandemic influenza A (H1N1) virus spread rapidly around the world, and even some patients developed certain severe complications. We reported one case of hemophagocytic lymphohistiocytosis (HLH) induced by severe pandemic influenza A (H1N1) virus infection. A 17-year-old girl had acute onset of fever, dry cough, rhinorrhea, and sore throat Her family members and close friends also had the similar symptoms. Anti-infection treatment with penicillin was given after 8 days of the onset of symptoms in the local hospital, and her chest radiograph showed consolidation of the left lung. Then, she was sent to the People's Hospital of Guizhou Province in China and endotracheal intubation were underwent on the ninth day for acute hypoxic respiratory failure. She was diagnosed with HLH induced by severe pandemic influenza A (H1N1) 2009 virus. Oseltamivir, steroids, immunoglobulin, and plasmapheresis were given immediately after admission. After being treated in the People's Hospital of Guizhou Province for 16 days, she was discharged. This experience shows that HLH may be a life-threatening complication for severe pandemic influenza A (H1N1) 2009 virus infection and responds well to therapy.

## 1. Introduction

The first case of human infection with pandemic influenza A (H1N1) 2009 virus was identified in Mexico in April 2009. The first three cases of confirmed pandemic virus in China were reported in May 2009 [1]. 

Hemophagocytic lymphohistiocytosis (HLH) is a rare clinical-pathological condition, which is characterized by the activation of the mononuclear phagocyte system, along with the hemophagocytosis in the reticuloendothelial systems. The rare case of HLH induced by pandemic influenza A (H1N1) 2009 virus infection was reported, and it was successfully treated. 

## 2. Case Presentation

After catching a cold, a 17-year-old, 160 cm height, 40 kg weight, previously healthy girl had acute onset of fever, fatigue, sore throat, and rhinorrhea for 8 days and dyspnea for 3 days. Then, she was sent to the emergency department of a local hospital for medical help on January 6, 2010. Though conventional medications were given to the patient, no obvious symptomatic relief was obtained. The next day, she was sent to the emergency department of the People's Hospital of Guizhou Province on account of persistent fever. 

**Figure 1:**
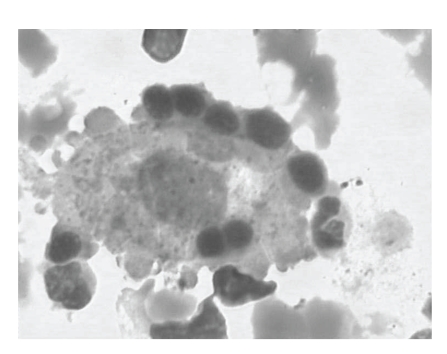
Bone marrow smear showed clasmatocytes engulfing blood cells (Wright's staining, 10 × 100).

On examination, she was conscious and well oriented. Her temperature was 40°C–40.5°C, respiratory rate was 38 per minute, pulse rate was 144 beats per minute, blood pressure was 102/70 mmHg, and oxygenation saturation was 90% while she was given an oxygen mask with 8 liters per minute oxygen flow. Auscultation revealed bilateral rales and regular heart sounds, and the spleen tip was soft and barely palpable under the left rib margin. Computed tomographic (CT) scanning showed consolidation and ground-glass opacities of left lung. Laboratory tests revealed white blood cell (WBC) 2.17 × 10^9^ cells/L, hemoglobin 116 g/L, platelet 46 × 10^9^ cells/L, and C-reactive protein (CRP) 158.30 mg/L (Table 1). Arterial oxygen tension/inspired oxygen fraction ratio (PaO_2_/FiO_2_) was lowered to 94.2, so she received intubation and mechanical ventilation and then transferred to the intensive care unit (ICU). On January 8, WBC count and CRP level were slightly increased, while hemoglobin and platelet obviously decreased. Triglycerides were 3.13 mmol/L, NK-cell activity was low (determined by flow cytometry, normal range was 7~40%) [2], repeated blood cultures were negative, ultrasound investigation revealed splenomegaly (the length is 13.1 cm, and the expected length is 11-12 cm), and her bone marrow smear showed that histiocytes were engulfing blood cells (Figure 1). Real-time reverse transcriptase-polymerase chain reaction (RT-PCR) of throat swab specimens for pandemic influenza A (H1N1) 2009 virus was positive, which was performed in the laboratory of the Guiyang Centers for Disease Prevention and Control. She was diagnosed with HLH induced by severe pandemic influenza A (H1N1) 2009 virus. 

She was treated with high-dose oseltamivir (150 mg twice daily, for 5 days, following 75 mg twice daily, 2 days), high-dose immunoglobulin (20 g once daily, 5 days), plasmapheresis (every other day, 2 days), and methylprednisolone (40 mg twice daily, 6 days). She was extubated on January 13. The next day, the retest of throat swab specimens for pandemic influenza A (H1N1) 2009 virus by RT-PCR was negative. On January 19, her temperature dropped to 38.2°C, while platelet increased to 108 × 10^9^ cells/L and triglycerides decreased to 1.20 mmol/L. All the results suggested that her HLH had already has improved, indicating that HLH might be induced by H1N1 infection. She was transferred to the respiratory department for left small pleural effusion after her fever has improved and left lung consolidation resolved. On January 23, the patient almost recovered and was discharged. 

## 3. Discussion

This case was diagnosed as pandemic influenza A (H1N1) 2009 virus infection by RT-PCR at the 10th day after symptom onset. Soon after, she had several conditions such as fever, splenomegaly, cytopenias, hypertriglyceridemia, haemophagocytosis in her bone marrow, and low NK-cell activity. According to the diagnostic criteria made by Histiocyte Society, HLH is defined by the presence of at least five of the following criteria: (1) fever, (2) splenomegaly, (3) bicytopenia, (4) hypertriglyceridemia and/or hypofibrinogenemia, (5) haemophagocytosis, (6) low/absent NK-cell activity, (7) hyperferritinemia, and (8) high-soluble interleukin-2 receptor levels [3]. The last two parameters cannot be determined in our hospital, but the present case met the former 6 criteria, so she could be diagnosed with HLH. Since this case did not have other risk factors which may be associated with HLH except for pandemic 2009 H1N1, she was finally diagnosed with HLH induced by severe pandemic 2009 H1N1 virus infection.

HLH is a life-threatening condition, which can be classified into genetic HLH and secondary HLH. Both types can occur at any age and are associated with increased mortality. Autosomal gene defects can cause genetic HLH or other immune deficiencies, such as Chediak-Higashi syndrome, X-linked lymphoproliferative syndrome, and Griscelli syndrome [4]. Secondary HLH is associated with exogenous agents (including viruses, bacteria, fungi, parasites, and toxins), endogenous products (including those resulting from tissue damage, metabolic products, and free radical stress), rheumatic diseases, and malignant diseases [5]. HLH induced by viral infection was first described in 1979 [6]. Apart from H1N1 virus, others such as human immunodeficiency virus (HIV), cytomegalovirus (CMV), and Epstein-Barr virus (EBV) have been reported to trigger the disorder [7–9]. 

 The pandemic H1N1 virus is a triple-reassortant influenza virus containing genes from human, swine, and avian influenza viruses. Unlike typical seasonal flu patterns, the pandemic H1N1 virus caused high levels of summer infections in the northern hemisphere and then even higher levels of activity during cooler months in this part of the world [10]. The study launched by Cao et al. showed that 21.4% of the patients had leukopenia, of which 68.1% of adults and 92.3% of children had lymphopenia in China. Delayed treatment with oseltamivir (≥48 hours) is an independent risk factor for a prolonged period of RT-PCR positive, but none of the patients reported had thrombocytopenia [11]. The present case had a sharp drop in platelets, and the possible reason was that it was too late (9 days, far more than 48 hours after the onset of symptoms) for her to receive oseltamivir treatment. The patient had developed HLH induced by H1N1 virus, so this is the reason why she was in critical condition. Treatment with high-dose oseltamivir was necessary for her life-threatening situation. 

There may be several ways to treat secondary HLH. High-dose corticosteroids are commonly used due to the inflammatory nature of the condition. Intravenous immunoglobulin can enhance humoral immunity as an immunomodulator, which appears to be more effective in infection-mediated HLH. Plasmapheresis, which was first used to further decrease the cytokine storm in 1982, [12], was an effective therapeutic tool for HLH. Apart from cytotoxic chemotherapy, the comprehensive therapy mentioned above had been carried out, and a rare hematological complication of pandemic H1N1 virus infection was successfully cured.

In conclusion, if febrile patients with pandemic H1N1 virus infection are associated with cytopenia, a bone marrow aspiration is recommended. Once confirmed as HLH cases, they should be given directed therapy as early as possible. 

## Figures and Tables

**Table 1:** Laboratory test results of the case on different days.

Variable	01/07/2010	01/08/2010	01/14/2010	01/20/2010
Days in the People's Hospital of Guizhou Province	1	2	7	13
Pandemic influenza A (H1N1) 2009 virus (RT-PCR)	—	Positive	Negative	—
White blood cell (cells/L)	2.17 × 10^9^	3.82 × 10^9^	13.24 × 10^9^	8.88
Differential count (%)				
Neutrophils	22.5	74.2	86.5	68.4
Lymphocytes	76.5	7.9	8.8	24.1
Red blood cell (cells/L)	4.69 × 10^12^	2.92 × 10^12^	3.62 × 10^12^	4.71 × 10^12^
Hemoglobin (g/L)	116	75	103	127
Platelet (cells/L)	46 × 10^9^	19 × 10^9^	102 × 10^9^	122 × 10^9^
C-reactive protein (mg/L)	158.30	175.50	16.20	9.2
Albumin (g/L)	30.3	18.8	38.1	36.9
Triglyceride (mmol/L)	—	3.13	1.76	1.68
Creatinine (umol/L)	190.7	332.8	82.6	66.1
Urea nitrogen (mmol/L)	8.4	21.3	10.8	6.5
Alanine transaminase(u/L)	52	56	32	14
Direct bilirubin (mol/L)	12.7	11.9	5.9	5.0
NK-cell activity	—	2%	7.5%	20%
Blood cultures	Negative	Negative	Negative	Negative

“—”Denotes no data.
